# Linking chemical surface water monitoring and pesticide regulation in selected European countries

**DOI:** 10.1007/s11356-024-33865-y

**Published:** 2024-06-12

**Authors:** Simon Spycher, Dennis Kalf, Joost Lahr, Mikaela Gönczi, Bodil Lindström, Emanuela Pace, Fabrizio Botta, Nolwenn Bougon, Pierre-François Staub, Kristina L. Hitzfeld, Oliver Weisner, Marion Junghans, Alexandra Kroll

**Affiliations:** 1 Daten Spycher GmbH, 8057 Zürich, Switzerland; 2 Rijkswaterstaat, Ministry of Infrastructure and Water Management, PO Box 17, 8200 AA Lelystad, the Netherlands; 3 National Institute of Public Health and the Environment, PO Box 1, 3720 BA Bilthoven, the Netherlands; 4 Department of Aquatic Sciences and Assessment, SLU Centre for Pesticides in the Environment, Swedish University of Agricultural Sciences, P.O. Box 7050, 75007 Uppsala, Sweden; 5 Italian Institute for Environmental Protection and Research (ISPRA), 00144 Rome, Italy; 6 Unit of Pesticidovigilance, ANSES, Maisons-Alfort, France; 7 French Biodiversity Agency–OFB, 94300 Vincennes, France; 8 German Environment Agency (UBA), 06844 Dessau-Roßlau, Germany; 9 Swiss Centre for Applied Ecotoxicology, 8600 Dübendorf, Switzerland

**Keywords:** Pesticides, Plant protection products, Chemical monitoring, Surface water, Monitoring strategy, Pesticide regulation, Environmental risk assessment

## Abstract

The progress in chemical analytics and understanding of pesticide dynamics in surface waters allows establishing robust data on compounds with frequent exceedances of quality standards. The current chemical, temporal, and spatial coverage of the pesticide monitoring campaigns differs strongly between European countries. A questionnaire revealed differences in monitoring strategies in seven selected European countries; Nordic countries prioritize temporal coverage, while others focus on spatial coverage. Chemical coverage has increased, especially for non-polar classes like synthetic pyrethroids. Sweden combines monitoring data with agricultural practices for derived quantities, while the Netherlands emphasizes spatial coverage to trace contamination sources. None of the EU member states currently has established a process for linking chemical surface water monitoring data with regulatory risk assessment, while Switzerland has recently established a legally defined feedback loop. Due to their design and objectives, most strategies do not capture concentration peaks, especially 2-week composite samples, but also grab samples. Nevertheless, for substances that appear problematic in many data sets, the need for action is evident even without harmonization of monitoring programs. Harmonization would be beneficial, however, for cross-national assessment including risk reduction measures.

**Supplementary information:**

The online version contains supplementary material available at 10.1007/s11356-024-33865-y.

## Introduction

Experience with chemical monitoring of pesticides in surface waters has been accumulating over the past 50 years starting with linking major environmental incidents with environmental concentrations (see examples in Tenney and Higgins (1972)). Endosulfan, e.g., was detected via gas chromatography (GC) following a fish kill in the River Rhine (Greve and Wit (1971) cited in Tenney and Higgins (1972)). In order to improve detection limits, GC and liquid chromatography (LC)-based quantification methods (Brouwer et al. 1995; Chen and Wang 1996; Dinelli et al. 1996; Slobodník et al. 1995; Tadeo et al. 1996) as well as extraction methods (e.g., reviewed by Eisert and Pawliszyn 1997; Matisová and Škrabáková 1995)) were increasingly used and were further refined and established in the following two decades (e.g., Huntscha et al. (2012)). Around 2010, the introduction of high-resolution mass spectrometers (HRMS) enabled the simultaneous screening of a large number of substances and revolutionized throughput and precision. HRMS can now provide comprehensive monitoring datasets such as for the River Rhine (Ruff et al. 2015) or selected rivers in Switzerland, Spain, the Netherlands, or the USA (Baas et al. 2016; Bradley et al. 2017; Moschet et al. 2014; Pinasseau et al. 2019; Pitarch et al. 2016). Brack et al. (2018) estimated that “it seems to be not unrealistic that in near future chemical monitoring of surface waters, sediments and biota can include 1000 and more target chemicals with limited additional costs and efforts”. Further possibilities arise from the application of suspect screening (Moschet et al. 2013; Singer et al. 2016) and from the very challenging but potentially rewarding field of non-target screening (Anliker et al. 2020).

Chemical monitoring of surface waters became obligatory for EU member states when the Water Framework Directive (WFD, 2000/60/EC) came into force in 2000. The WFD requires member states to measure chemicals defined as priority substances (Annex 1 of the Environmental Quality Standards Directive 2013/39/EU (EQSD), 45 substances) and chemicals on the surface water Watch List that should be renewed every 2 years. Through the Watch List, monitoring data is obtained to determine the risk of selected substances and whether environmental quality standards (EQS) should be set at the EU level (Loos et al. 2018). An EU-wide risk assessment is performed for priority substances based on the EQS listed in Annex II of the (Directive 2013/39/EU) to determine the chemical status of surface waters together with other parameters. A good chemical status is achieved when the EQS are complied with. Updated versions of the WFD and EQSD were recently open for public consultation, including the updated list of priority substances and EQS (24 new substances, one group of substances (PFAS), and a maximum level for the total pesticide concentration were added). Additionally, member states have to set national EQS for substances posing a risk at the national level, i.e., river basin-specific pollutants (RBSPs) that are included in the determination of the ecological status of surface waters in the current version of the WFD. Besides industrial chemicals, pesticides are an important group of substances regulated by the current EQSD. In Switzerland, the WFD does not apply. Substances posing a risk in Swiss surface waters, including many pesticides, are regulated with acute and chronic aquatic quality standards in Annex II of the National Water Protection Ordinance.

For the authorization of pesticides, EU and Swiss legislation distinguishes between active substances (a.s.) in plant protection products (PPP), a.s. in biocidal products (further referred to as biocides), and a.s. in human or veterinary medicines. The present article focuses on the monitoring and regulation of a.s. used in PPP. The pathways to surface waters of a.s. in PPP and biocides and also their regulation differ. Active substances from PPP mainly enter surface waters directly from the site of use, e.g., in crops, whereas a.s. from biocides largely enter surface waters via treated wastewater, but overlapping aspects will be discussed.

According to Art. 21 of the regulation for placing on the market of PPP (1107/2009/EC; PPPR), exceedances of EQS of priority substances can, in principle, be used to re-evaluate approval of PPP a.s. on the EU level as this would indicate that part of the objectives of the WFD (reaching good chemical status, i.e., compliance with the EQS) are not met. National authorizations of PPP may be reviewed on the same grounds (Art. 44 1107/2009/EC). However, based on the list of priority substances still in force, out of the 20 pesticides listed, 13 are already banned for application in European agriculture. Surface water monitoring data were not considered in any of these decisions. The final renewal report for cypermethrin mentions, however, that the substance was added to the list of priority substances in 2013 and that “Member States shall consider to set appropriate monitoring requirements when granting authorizations in accordance with Article 6(i) of the PPP Regulation” (EC 2021). Recently, the Swiss Water Protection Act and Water Protection Ordinance have been updated with a direct link between chemical monitoring data and PPP and biocides authorization as discussed further below. Experience is not yet available as the implementation is too recent. Within the national monitoring, chemicals are selected via a prioritization process based on their occurrence from modeling and specific and comprehensive monitoring campaigns. These comprehensive studies with both a high number of substances and a high temporal coverage showed first that the EU priority substances only cover a small fraction of the most relevant compounds (Moschet et al. 2014) and, second, that a limited set of substances currently included in the Swiss national monitoring covers a large fraction of the risks (Spycher et al. 2018). Taking the measurement campaign from the year 2012 in Swiss rivers as an example (Moschet et al. 2014), 48 of the 50 most frequently detected pesticides were not on the list of priority substances. In the EU, these 48 non-priority substances do not have to be monitored and do not contribute to the determination of the chemical status. As summarized by Brack et al. (2018), there is evidence that the regulation of individual chemicals as priority substances often results in a replacement with non-regulated substances. Apart from the limited number of analytes prescribed by the WFD, further potential reasons for underestimation of environmental risks of pesticides in surface waters have been discussed by Weisner et al. (2022). In particular, timing and site selection of the minimum monitoring requirements seem to be unable to adequately capture the periodic occurrence of pesticides from agriculture, and the level of protection and availability of regulatory thresholds are deemed insufficient to ensure a good ecological status. As a consequence, EU member states complement the mandatory WFD monitoring with regional or national surface water monitoring programs in order to grasp the actual risk posed by pesticides.

The WFD covers all waters including inland surface waters and sets out specific requirements for chemical monitoring. It distinguishes surveillance, operational, and investigative monitoring. Surveillance and operational monitoring programs are generally mandatory, while investigative monitoring is mandatory under specific circumstances. For rivers, the WFD requires monitoring of both upstream and downstream sites, as well as sites that represent different ecological conditions, such as natural or heavily modified water bodies. Sampling frequencies shall take account of the variability in parameters; the selected sampling times shall minimize the impact of seasonal variation. Monitoring during different seasons may be necessary to achieve this objective for chemicals which are not continuously entering surface waters. The minimum sampling frequency for priority substances in the frame of the obligatory surveillance monitoring is on a monthly basis and for RBSP on a quarterly basis. The type of sampling required is not prescribed, but recommendations (e.g., on grab sampling, event-driven, and time-proportional sampling) are given in Guidance Document No 7 “Monitoring under the water framework directive” (EC 2012) and Guidance Document No 19 “Guidance on surface water chemical monitoring under the water framework directive” (EC 2009). Hydrological and geological conditions vary substantially among the EU member states. With respect to PPP, inputs via runoff, drainages, or exfiltration from groundwater may vary despite similar size and stream order. Consequently, the Guidance Documents stress that national and regional adaptations may be necessary to meet the monitoring objectives (EC 2009, 2012). Yearly national implementation reports, including chemical monitoring under the WFD, are available from the European Commission (EC 2023). Streams in agricultural areas with comparatively low dilution of PPP input are most sensitive to risks posed by PPP but often are the least sampled, as examples from Switzerland and Germany show (Brinke et al. 2017, Munz et al. 2012). Against this background, our contribution focusses on surface waters with special consideration of streams, canals, and ditches in agricultural areas. We consciously excluded standing waters due to the variable influence of groundwater, although acknowledging that ponds are especially important as habitats for amphibians.

Monitoring the input of PPP into streams, canals, and ditches poses particular challenges as opposed to monitoring of urban contaminants which are mostly emitted by point sources into larger streams in an often continuous manner. For industrial chemicals, the situation can be more complex with potentially strong emission peaks from industrial production sites (Anliker et al. 2020). PPP emissions are mostly due to diffuse sources (Leu et al. 2010) with the particular challenge of peak concentrations during application seasons that vary in three dimensions: chemicals emitted, time of emission, and site of emission (Wolfram et al. 2021). While monthly grab samples may provide a reasonable overview of continuously emitted contaminants in a larger stream at a defined sampling site, however, the same strategy will most likely miss peak concentrations and variability of PPP emissions in agricultural areas (Leu et al. 2004; Liess et al. 2021).

In the past 15 years, several groups of authors have approached these issues providing overviews of monitoring strategies in the countries represented in the Nordic Council (2007) and the Northern Zone as defined by the PPPR, 1107/2009/EC (Stenrød et al. 2016) as well as comprehensively analyzing all monitoring data reported under the WFD between 2001 and 2015 (Wolfram et al. 2021). According to an analysis by the European Environmental Agency (EEA) of monitoring data reported between 2013 and 2020, at least one pesticide was detected above its effect threshold at 22–32% of all monitoring sites on rivers (small, medium, large) in each year of assessment despite the limitations of the WFD minimum monitoring requirements (EEA 2022).

Against this background, the present study has two main goals. First, to give an overview of up-to-date key indicators characterizing surface water pesticide monitoring strategies in different European countries. The comparison also includes organizational aspects like funding and data accessibility. The monitoring strategies are contextualized regarding their suitability for different purposes. Second, to describe the available links between pesticide monitoring and regulatory risk assessment and to provide case studies on how consequences can be drawn from monitoring in light of the limited possibilities legally specified by the PPPR (1107/2009/EC).

## Methods

Data collection was performed via a detailed questionnaire as relevant information is often found in the grey literature in national languages. A survey was conducted with the questionnaire available in the supplementary information (Annex 1) to retrieve information on national chemical monitoring programs for surface waters and on the use of chemical monitoring data for the authorization of PPP. The survey was conducted in collaboration with six EU member states representing all three zones with respect to EU PPP authorization: Denmark (DK) and Sweden (SE) in the Northern Zone, France (FR) and Italy (IT) in the Southern Zone, Germany (DE) and the Netherlands (NL) in the Central Zone, as well as Switzerland (CH) which is not a member of the EU but has aligned the approval of a.s. to the decisions taken in the EU as defined in the CH Plant Protection Product Ordinance. The WFD does not apply in CH but the national Water Protection Act and Water Protection Ordinance.

The results of the survey were discussed in an online workshop with experts from the aforementioned countries to elucidate specific differences in chemical monitoring and regulation between countries and to identify the advantages and limitations of the different approaches.

## Goals and structure of pesticide monitoring programs

### Goals of monitoring programs

The national monitoring programs described here have been designed with two main aims: the identification of exceedances of regulatory thresholds on the one hand and the assessment of the success of implementing risk reduction measures on the other hand. In the EU member states, not all of the programs or all parts of the programs are defined as monitoring under the WFD but often fall within the definition of surveillance monitoring according to the WFD (provide a general overview of the state of the water body to identify any emerging issues and to assess whether the measures taken to protect and improve water quality are effective). The sampling strategies vary strongly from country to country concerning chemical, temporal, and spatial coverage and also the type of threshold concentrations used to assess measured concentrations. The organization of the programs, likewise, varies. SE and NL have established monitoring networks specifically for pesticides or even specifically for PPP in agriculture next to the general WFD monitoring, while others operate a general chemical monitoring for pollutants run by the federal states (DE), regions (IT), or by the water agencies at the scales of the main hydrographic districts (e.g., FR, with some agencies also running some extra pesticides monitoring, additional to those legally requested for the WFD waterbodies assessment). The national solutions for pesticides are therefore a continuum from fully separate pesticide monitoring programs to programs integrated into the WFD monitoring. In CH, national legislation applies, and the cantons and the federal government share responsibility.

### Sampling strategies

#### Chemical coverage

The number of a.s. approved for PPP in European countries is continually changing but currently lies between 200 and 300 synthetic-organic compounds. For example, in Germany, sales data for the year 2020 published by the German Federal Office of Consumer Protection and Food Safety (BVL) list a total of 287 a.s. with reported sales, 229 of them being synthetic-organic compounds (inorganic compounds, organisms, and non-specific organic compounds like acetic acid or fatty acids excluded) (BVL 2021). Forty-three compounds had a usage below 1 t, and thus the area treated is rather limited even for a.s. which are applied at small amounts per hectare. Thus, the number of a.s. to monitor presents a considerable challenge for analytical chemistry labs, but it can be reduced if the compound selection is prioritized by environmental relevance. As an example, SE developed a system for including new substances in the analyses based on adding up six parameters, each graded 0 to 10, resulting in a maximum of 60 points. The included parameters are (1) EQS: the lower the EQS, the higher the number of points (more important to include); (2) WFD: substances already included in the legislation receive more points (priority substances = 10 points; RBSP = 9 points (Havs-och vattenmyndigheten (2019)); WFD Watch List = 8 points); (3) DT50: the longer the degradation time, the higher the number of points; (4) K_foc_: (more points if easily leached); (5) Amount used: the higher the amounts applied in the national monitoring catchments, the higher the number of points; (6) Treated area: more points if larger surface of national monitoring catchments is treated. The list is updated on a yearly basis; in that way, all samples taken during 1 year are analyzed for the same a.s. Similar procedures for substance selection have been established in other countries.

In the countries with available information on the average number of a.s. measured per sampling location, this metric varies between 12 and 135 a.s. (Table 1). While SE has a fixed set of a.s. measured on all locations, most other countries have variable chemical coverage. NL, in particular, adapts the number of measured a.s. based on information on the crops grown in the catchment and the a.s. typically used on these crops, and thereby, they can substantially reduce the number of measured a.s. in catchments with limited pesticide usage and also make sure that the analyte spectrum is not outdated. Some countries like CH have a mandatory minimal set of a.s. which have to be measured in each sample (row 2 of Table 1). However, the mean number of measured a.s. is substantially higher as can be seen for the examples of CH and DE with 50 and 100% more a.s. measured per sample than required on the national level, respectively. The total number of a.s. with reported quantifications in a country, i.e., the number of compounds which were analyzed (but not necessarily detected) in at least one sample over the reporting period, can reach several hundred substances in, e.g., DE or IT, but sometimes includes a large number of pesticides which have been discontinued or banned for usage in agriculture.

**Table 1:** Chemical coverage in surveyed countries

	DK	FR	DE	IT	NL	SE	CH
Mean number of a.s. per sample	-	53	80^[2]^	81^[4]^	-	135^[6]^	62
Minimum number of a.s. required for national monitoring per sample	12^[1]^		67^[3]^	-	-	135^[6]^	40
Number of a.s. with reported quantifications per country	-	229	246^[2]^	398^[4]^	182^[5]^	135^[6]^	158^[7]^

Notes and source unless from survey questionnaire:

^[1]^Only information on PPP required by WFD given

^[2]^Brinke et al. 2017, Section 3.4.3, and Table 4. Value in Table 4 includes a limited number of metabolites. Both values include a high number of compounds that were not approved anymore

^[3]^Weisner et al. (2022) (active substances which are RBSP)

^[4]^(ISPRA 2022), p. 19. Includes a relatively high number of pesticides which are not approved anymore and also a limited number of metabolites

^[5]^(Deltares 2020). Number of a.s. quantified determined by pesticide usage on the corresponding catchment and therefore variable

^[6]^The analytical list in SE includes an additional 15 metabolites, hence totaling 150 a.s

^[7]^Only samples of routine monitoring sites (NAWA TREND) and not special investigations (NAWA Spez)

Until recently, pyrethroids represented the biggest gap in chemical monitoring since EQS or other effect-based threshold values are generally below the limit of detection. From an ecotoxicological perspective, improving analytical methods is thus necessary. This gap is now gradually closing in some countries due to improved analytical methods with limits of quantification now falling in the picogram per liter range (Rösch et al. 2019). However, it will take some time until this gap can be closed in all countries and thus, there still is a substantial number of pyrethroids and also some other insecticides like pyriproxyfen which might have effects at concentrations well below the limits of quantification (LOQs) common in routine monitoring. The extent of these “invisible” risks was evaluated in an analysis in NL which for 2020 lists a total of 54 a.s. with LOQs above the EQS or other effect-based thresholds (Deltares 2021). On the other hand, a recent evaluation of routine monitoring data in CH showed that for insecticides approved for use in PPP in the year 2022, only the LOQ of deltamethrin lies consistently above its EQS. The LOQs of cypermethrin and lambda-cyhalothrin are sometimes above their quality criterion and sometimes below (Daouk et al. 2022) with the LOQ-variability caused by matrix effects and differences between laboratories. Compounds like tefluthrin for which quality criteria had not yet been derived could not be evaluated. The examples shown above make clear that comparison between countries requires accounting for differences in LOQs. The other gap consists of very polar compounds and compounds which are rapidly degraded, but based on the available fate modeling and ecotoxicological data, they present lower risks.

Some countries like DE, FR, and IT report high total numbers (> 200) of quantified compounds with a limited number being metabolites. However, a substantial portion of the a.s. are not approved anymore. Some of them, e.g., DDT, have not been used for decades. Furthermore, many of these compounds were detected only on a few occasions. In conclusion, maximizing the number of analyzed a.s. appears less important than achieving sufficient coverage in terms of usage and/or risks. Analytical methods are now able to cover the most relevant proportion of pesticides. However, when it comes to the practical and affordable implementation in routine monitoring, there are still gaps to be closed, especially for synthetic pyrethroids where EQS are often below the LOQ.

#### Temporal coverage

**Fig. 1:**
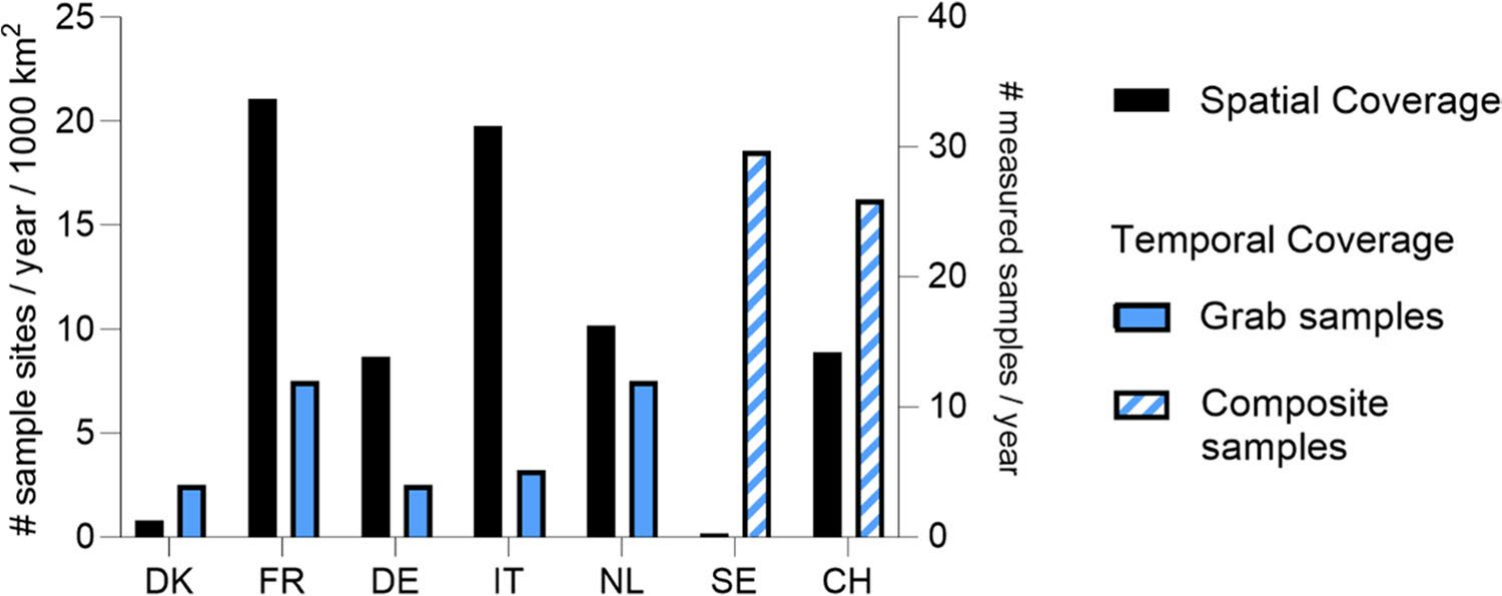
Average number of samples per site and year and number of sampling sites per year per 1000 km^2^ of cropland, i.e., arable land and permanent crops. Note that the bars may, in some cases, represent rough estimates or proxies such as averages for reasons of simplification (details in Table A3)

The number of samples per site and year varies considerably between the surveyed countries. SE and CH, the two countries using composite samples, take on average more than 20 samples per site per year with the sampling duration ranging from 2 weeks to 3.5 days. On the other end of the spectrum, countries like DK and DE limit the number of samples per site to the minimum requirement of the EU WFD of four yearly samples for non-priority substances (Fig. 1).

Figure 1 depicts the total number of samples per site per year analyzed ultimately in the lab. However, as composite samples taken in SE and CH consist of subsamples taken every 90 or 45 min, respectively, they are made up of up to 100 to 200 subsamples a week. Thus, the actual temporal coverage is substantially higher and covers the whole year. As a consequence, these sampling strategies integrate close to all inputs into the streams, i.e., inputs via run-off but also inputs via point sources. However, such strategies can also hide episodic short-living peaks, which may be diluted (la Cecilia et al. 2021). The four countries working with grab samples were analyze between four and twelve samples per site per year usually in fixed intervals. Such sampling strategies tend to miss short-term peak concentrations caused by varying inputs via point sources or run-off during rain events and thereby underestimate the risks posed to aquatic organisms.

Passive sampling has been tested at a large scale in France for the purpose of WFD monitoring (Mathon et al. 2022). This led to the official acceptance of this sampling method as a complementary technique for the national regulatory WFD monitoring, but it has not yet been operationally implemented by the water agencies. Also, within the proposal to revise the EU Water Framework Directive (EC 2022), passive sampling has been included as a monitoring option, “in particular for screening purposes, on the condition that those sampling methods do not underestimate the concentrations of pollutants for which environmental quality standards apply.”

#### Spatial coverage and inclusion of small water bodies

The spatial coverage was calculated by dividing the number of sampling sites per year by the agricultural land most relevant for pesticide usage, namely the sum of arable land and permanent crops also referred to as cropland (Table A1 in the Supporting Information). Agricultural land used for meadows and pastures (grassland) was considered less relevant. The rationale is that grassland is treated much less and with a much smaller group of compounds than land used for crop production. The assumption is corroborated by usage data for Switzerland showing that grassland is treated 10 to 100 less frequently (de Baan et al. 2015) and by the comprehensive surveys in the UK showing that 95% of the grassland surface of 11 million ha received no treatment and the other 5% being treated with an average of 1.1 spray rounds per year (Ridley et al. 2021). The distinction between cropland and grassland greatly affects the calculated surface used to derive the spatial coverage. For countries like CH, where meadows and pastures make up a large fraction of the agricultural land, the spatial coverage can differ by up to a factor of five depending on the metrics used, while for countries like DK with large fractions of arable land, the difference is less than 10%. The crop areas published by FAO-stats were used for obtaining the necessary information and comparing the seven countries (FAO 2022) (values given in Table A1 of the Supporting Information and a comparison of FAO-data with national statistics given in Table A2).

The spatial coverage differs strongly between the countries (Fig. 1). It is inversely related to the temporal coverage in some cases, e.g., SE has a high temporal and low spatial coverage, although additional targeted screenings to enhance the spatial coverage are done regularly, and the number of Swedish sites does not include those from WFD monitoring. CH has both a high temporal and a fairly high spatial coverage.

Small water bodies are most affected by agricultural activities (Lefrancq et al. 2017; Spycher et al. 2018; Szöcs et al. 2017), but they are often underrepresented in the selection of sampling locations. An analysis of the DE monitoring locations showed that only 12% of the monitored catchments are smaller than 10 km^2^, although small streams make up for the largest share of the river network (Brinke et al. 2017). In their extensive and Europe-wide analysis of all WFD data sets recorded between 2001 and 2015, Wolfram et al. (2021) showed that small streams were underrepresented on a European level with the median catchment size of the WFD catchments being 238 km^2^. CH corrected its similarly skewed distribution of monitoring sites described in earlier studies (Munz et al. 2012) in the last years, and now, 40% of the CH catchments monitored for pesticides have a surface of less than 10 km^2^. In SE, small water bodies are well covered with the surface of the four smaller catchments ranging from 8 to 16 km^2^. It is important to note that there is no clear-cut definition of which size or stream order a stream can be considered small or very small. Some authors use the stream order according to Strahler (1952) and define all streams up to a stream order of 2 (Munz et al. 2012) or 3 (Lorenz et al. 2017), respectively, as small streams, while others set the limit based on the catchment area < 30 km^2^ (Brinke et al. 2017) or < 100 km^2^ (Lorenz et al. 2017) or on classes of hydrological metrics for discharge. Moreover, lowland countries like NL have many small waters like ditches and canals that are not characterized as streams because the current is relatively low.

#### Information on the origin of pesticides

Some a.s. are not only used for agricultural production but also as biocides, veterinary pharmaceuticals, and/or drugs for human health. An evaluation of the lists of PPP and biocides authorized in CH showed that as of January 2022, a total of 215 a.s. were registered for use in PPP and 135 for use in biocides with an overlap of 33 a.s. (inorganic a.s. and biocontrol methods like parasitic wasps or granuloviruses excluded) which corresponds to 15% and 25% of all a.s., respectively. In order to interpret monitoring results, it is thus important to have information on the usage within the catchments and the possible pathways by which pesticides reach surface waters. Information on the main usages helps to narrow down the possible sources and thus propose effective measures for reducing the inputs. Another possible source is amenity use of PPP, e.g., usage for golf courses, parks, traffic infrastructure, and also usage by non-professional users. The number of a.s. relevant for the latter usage can be narrowed down using information on authorized pre-formulated products. In SE, for example, only PPP with a low-risk profile are allowed for non-professional use. In DE, 57 a.s. are currently available in PPP authorized for non-professional use (BVL 2023a).

The share of agricultural and urban land use in the catchment is the most common indicator for the possible source of the measured pesticides. As an example, the catchments of small streams in SE have more than 85% arable land (and more than 70% cropland, respectively), very low to no urban land use, and no train tracks. The sites of the NL pesticide monitoring network (LM-GBM) have been carefully selected to minimize inputs from urban use or other non-agricultural sources. On many of the LM-GBM-sites, it is even possible to distinguish between dominant crop groups like flower bulbs, greenhouse horticulture, fruit cultivation, arable farming, and fodder crop production (maize and grassland). In general, data from catchments with predominant agricultural land use can be used to put monitoring data into perspective and minimize the overlap with other usages, e.g., application as biocides.

A common method to determine an influence of wastewater is to measure marker substances like artificial sweeteners or selected human pharmaceuticals (Table 2). This aspect is of importance for catchments which also have urban influences but are not relevant in catchments for which a clear domination of agricultural inputs is the evident form of land use like in NL and SE.

**Table 2:** Overview of measures to track the origin of detected pesticides

	DK	FR	DE	IT	NL	SE	CH
Measurement of WWTP markers	No	Partially	No^[2]^	NA	No^[4]^	No^[6]^	Yes
Comparison with PPP use data	Yes	Partially^[1]^	No^[3]^	No	Partially^[5]^	Yes^[7]^	Not yet
Comparison with other usages	No	No	No	No	Depending on location^[8)^	No	Not yet

^[1]^Work is underway to assess the relationship on a national scale

^[2]^Not for routine monitoring, but in project “Kleingewässermonitoring” (Liess et al. 2022)

^[3]^Partially done in the case study of project “Kleingewässermonitoring” (Liess et al. 2023)

^[4]^Influence of wastewater ruled out by selection of sampling location

^[5]^Most likely sources evaluated as part of emission reduction plans; emission modeling with breakdown to catchment scale

^[6]^No communal WWTP in the four monitoring catchments

^[7]^For the four monitoring catchments

^[8]^NB–the Dutch Pesticide Atlas has a tool that correlates measurements of pesticides to land use including broader types of crops

A particularly valuable source of information is PPP usage data on the catchment scale. In SE, such local usage data are collected since 2002 (Boye et al. 2019, Nanos et al. 2019, Sandin et al. 2018). In DK, survey data on usage are available. These are transformed into general DK use statistics of pesticides (MST 2023) but cannot be specifically used to compare to surface water monitoring data. CH has limited survey data from about 1% of the cropland (de Baan et al. 2015) and thus, like DK, it has no catchment-specific data but has decided to introduce a full registration of all applications for both PPP and biocides by 2025 (FOAG 2023). The other three countries do not dispose of information on PPP-usage at the catchment scale (Table 2), but countries like NL regularly adapt the list of compounds to measure based on the crops grown in the catchment and thereby make sure to measure a high percentage of actually applied compounds. The NL Pesticide Atlas (*Bestrijdingsmiddelenatlas* (Vijver et al. 2008)) also disposes of a tool wherein users can correlate measured pesticide concentrations and exceedance of EQS to various types of land use including different crop types. In a DE case study as part of the *Kleingewässermonitoring* (KgM, 4.3.1), PPP usage data from the agricultural areas in the upper reaches of the monitoring sites were compared to the measurements of the pesticide concentrations in the streams. In this way, measurements of a.s. could be directly linked to their agricultural use and even partially linked to individual PPP applications (Liess et al. 2023). The advantage of data on the catchment scale is that the pesticide concentrations and particular loads can be put into relation to the usage in the area making it easier to calculate loss rates, identify pathways into surface waters, and evaluate the efficiency of risk mitigation measures. No participating country has data on usage of biocides or veterinary drugs on the catchment scale.

For FR, purchased tonnage data of PPP a.s. are publicly available, based on the declarations of distributors. The purchaser’s postal code is communicated (except in areas with fewer than five potential purchasers), so that catchment scale information is theoretically identifiable, but local purchase data cannot be directly linked to local usage. These data make it possible to define the potential PPP pressure for a given area, an approach which has also been reported by some regional offices and local consortia in IT (e.g., Costa and Salandin (2022)), but unlike in FR, it is not generally available. Some basic information on usage can furthermore be derived from “Cultivation practices” surveys of the National Agency for Food, Environmental and Occupational Health Safety (ANSES) which are summarized in phytopharmacovigilance sheets (PPV) (ANSES 2023). Other potential uses of the a.s. are also listed (veterinary medicines, biocides), as well as the history of PPP authorizations for the a.s. by type of crop, list of authorized uses for PPP, and national sales data information.

On the national scale, all countries have information on the yearly sales of the a.s. used in PPP at its disposal, and DK, DE, NL, SE, FR, and CH publish the yearly sales on the level of the a.s. In accordance with the EU regulation on pesticide statistics (EC 1185/2009), all countries also conduct surveys on the usage of the PPP in different crops. Such data can be useful for a number of analyses such as the estimation of PPP emissions to air and surface waters in NL (www.emissieregistratie.nl) but cannot replace the information of usage data on the catchment level. Furthermore, in some countries, their coverage is rather limited, e.g., in DE with only about 100 farms (JKI 2023). Such data can nevertheless be valuable to derive some basic information, e.g., in which crops an a.s. are mainly used.

For biocides, only DK collects and publishes detailed data on biocide usage and sales (Miljøstyrelsen 2022). These are substantially lower for all a.s. which are also used as PPP. DE made reporting of biocide sales data mandatory and is expected to publish data from 2023 onwards (FMJ 2021). The same requirement has been implemented in the update of the Swiss Biocides Product Ordinance (*Biozidprodukteverordnung*, SR 813.12) coming into effect on January 1, 2024. NL will conduct a feasibility study in 2024.

### Threshold values for the assessment of chemical water quality

In most cases, effect-based threshold values derived according to the EU TGD for EQS (EC 2018) are used to assess measured pesticide concentrations. Only values that are legally binding are referred to as actual EQS. For substances not monitored as priority substances on the EU level but as RBSP on the national level, EQS are derived on the national level. These have partially been integrated into national legislation or are available from the national entity responsible for EQS derivation. In NL, assessment is based on a wider range of standards depending on the specific assessment: EQS, older national equivalent to EQS such as the Maximum Permissible Concentrations (MPC), and regulatory acceptable concentrations (RAC). SE mainly uses legally not binding water quality objectives (WQO) based on effect-based threshold values from EFSA conclusions for the national pesticide monitoring to enable WQO comparisons of all analyzed a.s. In addition, a toxicity index is calculated to estimate the additive mixture toxicity (Boye et al. 2019). However, EQS (EU priority substances and RBSP) are used in regional monitoring programs required by the WFD. The CH Water Protection Ordinance has defined national EQS for selected substances in surface water according to the TGD and a precautionary threshold value of 0.1 µg/L for all pesticides which have no established EQS. Overall, there are no fixed, regular intervals for updating national EQS and other effect-based thresholds based on new scientific knowledge.

### Organization, funding, and accessibility

Table 3 summarizes the organizational aspects of pesticide monitoring in the different countries participating in the survey. The organization of the monitoring campaigns is at the national level in DK. In SE, the main pesticide monitoring is at a national level, but there is also some regional monitoring according to WFD. In the other countries, it is devolved to the federal states (DE), cantons, and the federal government (CH) to water agencies defined by the major river basins (FR), water boards, and the authority for national surface waters *Rijkswaterstaat* (NL) or regions (IT) (Table 3). In the past, in CH, this situation led to major differences in the cantonal monitoring strategies (type of samples, number of samples, and compounds covered), but a harmonization effort brokered by the federal and cantonal authorities resolved these differences and helped establish a compulsory minimal set of substances to monitor the type and frequency of sampling, with many cantons measuring additional compounds depending on the situation in their catchments.

**Table 3:** Overview of organizational issues of monitoring campaign (*X*: yes, –: no)

Aspect		DK	FR	DE	IT	NL	SE	CH
Organizational responsibility		National level	Water agencies	Federal states	Regions	Water boards and national level	National and regional level^[1]^	Cantons and state
Costs covered by	Fees		100%					
	National government	100%				X	X	50%
	Federal states, regions			100%	X		X	50%
	Others, like water boards					X		
National database of measured concentrations		X	X		X	X	X	X
Public accessibility		–	X^[2]^	–	X^[3]^	X^[4]^	X^[5]^	Upon request

^[1]^Pesticide monitoring and specific-pesticide screening of, e.g., greenhouse areas is organized at the national level, WFD monitoring is at the regional level

^[2]^OFB (2023)

^[3]^ISPRA (2023)

^[4]^Vijver et al. (2008); www.bestrijdingsmiddelenatlas.nl

^[5]^(SLU 2023)

Monitoring campaigns are funded by public authorities in all countries except in FR where limited fees are collected by pesticide distributors. A part of these fees is specifically dedicated to diffuse pollution of agricultural origin, with a gradation by the hazard potential of the substance. On the other hand, the revenues of the tax based on the pesticide load indicator in DK are not used for monitoring purposes, as the revenues are redistributed to the farmers who benefit if they use products with a lower load indicator. Costs for monitoring are fully paid by the central government in DK, and this is also the main funding body in SE, while in CH, the costs are split between the national government (50%) and the cantons (50%). NL uses a different model, where sampling and analysis are paid for by the regional water boards, which also collect taxes, while the national government finances the monitoring in the larger national waters and the annual evaluation and the disclosure of the monitoring data in the online interactive Pesticide Atlas (Vijver et al. 2008).

The pesticide monitoring data for surface water as well as for groundwater are accessible via websites in FR, IT, NL, and SE (cf. footnotes of Table 3). In CH, they are available upon request. In general, swift publication of the data, e.g., at the end of the season, would be very helpful, for example, as a reference for PPP users and risk managers.

## Aquatic risk assessment for national authorizations of PPP

### Provisions at the member state level

In the EU, PPP a.s. are approved at the European level, whereas formulated PPP are authorized by the member states grouped in three geographical zones (Northern, Central, and Southern Zone). The corresponding environmental risk assessment for aquatic and sediment organisms is laid down in the EFSA “Guidance on tiered risk assessment for plant protection products for aquatic organisms in edge-of-field surface waters” (EFSA 2013). While data requirements for ecotoxicological effects and bioaccumulation are explicitly defined for a.s. approval and product authorization according to (EU) No 283/2013 and (EU) No 284/2013, exposure models are only described and recommended for the a.s. approval process. The EFSA guidance states that the FOCUS methodology (FOCUS 2023) is currently used for approval for a.s. at the EU level and in some Member States for PPP authorization, “but also different exposure assessment procedures may be used.” Further, Article 1 (4.) of EC 1107/2009 entitles member states to apply the precautionary principle “where there is scientific uncertainty as to the risks with regard to human or animal health or the environment posed by the plant protection products to be authorized in their territory.” This provision may justify stricter and/or modified assessments. Existing modifications communicated by the member states are described in the following. Within the Northern zone (NZ; Denmark, Estonia, Latvia, Lithuania, Finland, Sweden), a specific guidance for ERA has been published in order to define stricter approaches where necessary and to harmonize these as much as possible across the NZ (EU Northern Zone 2021; Stenrød et al. 2016). Additionally, DK has introduced exceptions as to the modeling parameters to be used nationally (DK EPA 2019). For the assessment of ecotoxicological effects, several specific requirements are foreseen in the NZ guidance document. Acute and chronic mixture toxicity is always required for all non-target organism groups regardless of the mode of action of the a.s. QSAR data is only accepted when it was already validated in the EU a.s. approval process. The refinement by using a detailed analysis of exposure profiles as indicated in the EFSA guidance document (Chapter 9.1; parts of Chapter 9.2 and Chapter 10.3.10) is not accepted. Only valid NOECs from mesocosm experiments are allowed (threshold option). In SE, farmers have to apply for permission to use PPP in drinking water protection areas. Evaluations are usually done with the model MACRO-DB, a field-scale pesticide leaching model (Lindahl et al. 2024).

NL has implemented several national procedures with individual decision trees: (1) for determining the risk of agricultural use of PPP for drinking water abstraction points, (2) for determining the risk of use of PPP on hard surfaces (pavements), (3) specific national drift spraying curves are used, and (4) for horticulture in greenhouses, i.e., farmers are obliged to purify their wastewater such that at least 95% of a.s. are removed. This 95% removal is also applied in the calculations for authorization purposes for use in greenhouses. IT and CH have not published national guidance documents.

In DE as in CH, exposure assessment for surface waters is performed separately for the exposure routes runoff, drainage, and spray drift using national exposure models.

The RACs derived from the submitted effects data for PPP authorization are publicly available in DE (list including critical effect data and assessment factors), CH (list without accompanying data), and NL (among others, within the online *Bestrijdingsmiddelenatlas* (Vijver et al. 2008)). IT and SE do not publish national RACs. The Swedish EPA publishes a list of non-legally binding WQO to be used in the evaluation of environmental monitoring results that are supposed to match the RAC published in the EFSA conclusions and may hence equal the RAC used in the authorization process, provided that the RAC is not based on further national refinements.

### Risk mitigation measures

The combination of risk mitigation measures and incentives for practitioners varies substantially between the member states. An overview is provided in Annex A2.2. We discuss selected aspects in more detail in the following. Riparian buffer strips are stretches along watercourses that are not used for agricultural purposes, as opposed to no-spray strips that may be planted with crops but not treated with PPP. Further, vegetated buffer strips can also be implemented in-field against surface runoff. The implementation of riparian buffer strips in PPP authorizations and water protection regulation is particularly interesting. DE has defined the goal that by 2018, 80% of the surface waters in sensitive areas should have permanent riparian buffer strips and 100% by 2023. However, this target had not been reached (60% in 2016), and now, it is not monitored anymore (national action plan for the sustainable use of plant protection products and *Deutscher Pflanzenschutzindex* (BMEL 2022)). According to the 2021 revision of the German Federal Ordinance on the application of PPP, riparian buffer strips of 5 or 10 m no-spray distance need to be respected when applying PPP (§4a *Pflanzenschutz-Anwendungsverordnung*). However, the law allows for adaptations on a federal-state level (*Länderöffnungsklausel*), resulting in differences in implementation and the existence of buffer strips. In CH, all water bodies are protected via a riparian buffer strip with a fixed distance to surface waters of 3 m being compulsory for all agricultural fields (*Chemikalien-Risikoreduktions-Verordnung*, SR 814.81). For Swiss farmers receiving direct payments (97% of all arable land, *Ökologischer Leistungsnachweis*, *ÖLN*), a distance of 6 m to surface waters is compulsory. This buffer is generally vegetated. Guidance is available for the correct measurement of the buffer strip (AGRIDEA 2017). The authorization of a PPP can impose further buffer strips (6, 20, 50, or 100 m depending on the risk) to reduce the risk of direct input to surface waters by spray drift. Further, vegetated buffer zones can also be implemented against run-off. These are only required if the slope of the field exceeds 2% (the same threshold is used in Germany) and if the distance to surface water is closer than 100 m. Further options are described in the instructions by the Swiss Federal Food Safety and Veterinary Office (BLV 2022). Inlet shafts of the road-storm drainage system constitute an indirect pathway whose inputs can exceed direct inputs to surface water (Schönenberger et al. 2022). Since 2023, the point system for the reduction of run-off, therefore, requires measures to reduce run-off on roads (*Anhang 1*, *Ziffer 6.1a.4 Direktzahlungsverordnung*, SR 910.13). In SE, surface runoff was excluded as a route of exposure in aquatic risk assessments of PPP intended for use (KEMI 2016). The main reason for this decision was that the R1 scenario in the PRZM-in-FOCUS model used for estimating pesticide transport via surface runoff has not been shown to be representative of Swedish conditions (Boye et al. 2012). Also, since the majority of arable soils in Sweden are tile drained, transport of pesticides via surface runoff may be regarded as a significant problem only in a minor part of arable land within the country. Consequently, buffer strips along watercourses have been excluded as a RMM in SE. However, there is a general legislation requiring 6 m spray-free in-field buffer strips along all lakes and streams and, there too, additional spray free distance depending on the wind conditions during application (Naturvårdsverket 2015). DK relies on the risk reduction measures suggested by EFSA (2013). Drift reducing nozzles need official documentation regarding their drift reduction potential (e.g., tested by Julius Kühn-Institut) to be accepted for use in DE and DK. If FOCUS Step 3 PECsw (predicted environmental concentration in surface waters) values are required to address the aquatic risk assessment in DK, a 2 m buffer zone must be stated on the label. In FR, the decree AGRG1937165A (of December 27, 2019, amending the decree of May 4, 2017) indicates that the width of the riparian buffer strips needs to be adapted to the PPP. A width or possibly several widths can be defined for the authorization of PPP based on the intended uses (5, 20, 50, or, if necessary, 100 m or more). The width can be reduced from 20 to 5 m or from 50 to 5 m, subject to compliance with the following conditions: (1) presence of a permanent vegetation system of at least 5 m width along the edge of the water points with shrubby plants. The height of the hedge must be at least equivalent to that of the crop. (2) implementation of officially accepted means (as described in the Official Bulletin of the French Ministry of Agriculture) to reduce drift or exposure to spray drift for aquatic environments. Any measure chosen shall make it possible to reduce the risk to the aquatic environment by at least three times compared to the normal conditions of use of the PPP. The use of PPP in the vicinity of water points must be carried out by respecting the untreated zone indicated in the marketing authorization or on the label. In case untreated zones are not defined, a generic untreated zone of at least 5 m needs to be respected. In NL, the “Implementation Programme for the Vision for the Future of Plant Protection 2030” (*Uitvoeringsprogramma Toekomstvisie Gewasbescherming* 2030) contains quantitative goals in relation to water quality. Riparian buffer strips are legally binding.

Conservation farming uses crop rotation, maximum soil cover, and conservation tillage (≥ 30% of the soil surface left is covered with crop residue, after planting, to reduce soil erosion by water) as tools to improve soil structure, limit erosion, and also run-off. This approach is only accepted as RMM in CH and DE. In CH, currently, 30% of arable land is dedicated to conservation tillage. Most common is mulch-till followed by strip-till. There are no specifications on the percentage of crop residues. The depth of soil disturbance and the type of machinery is specified (BLW 2021). In DE, there is no specification other than the field may not be tilled with a mouldboard plow (Klein et al. 2023).

The verification of correct implementation of RMM likewise varies between the member states. In FR, verification is the responsibility of state agencies, with a split between the regional entities of the French Office for Biodiversity (OFB) and the Regional Directorate for Food, Agriculture and Forestry (DRAFFs) depending on the measure. In SE, RMM implementation is verified in the frame of the controls of the farms by the municipal authorities. In DE, an annual plant protection control program is defined that is executed by the federal states, and a yearly national overview is published (BVL 2014, 2023b). The controls include verification of RMM. The cantons of CH are responsible for the control of the implementation of RMM. In NL, RMM that are prescribed on the label of PPP and/or laid down in national regulations are subject to surveillance and enforcement by the Netherlands Food and Consumer Product Safety Authority (NVWA) and the water boards.

#### Case study: emission reduction plans in the Netherlands

In NL, a specific national monitoring network for PPP (LM-GBM) with 106 monitoring sites has been established in 2014 in order to plausibly relate threshold concentration exceedances with the use of PPP within seven groups of crops. The Dutch government publishes results of the national chemical monitoring on a yearly basis (Vijver et al. 2008). The results are then discussed with the water boards in a consultation. All stakeholders including the public are informed about a.s. that are considered problematic based on these results. Active substances that exceed threshold levels the most are prioritized for the definition of emission reduction plans (ERP). These have been initiated due to the fact that Art. 44 of 1107/2009/EC specifically only refers to priority substances when stating that PPP use should not conflict with the aims set in the WFD. ERP are annually agreed on and evaluated by the government and the authorization holders. The measures set out in the ERP can be aimed at tightening the authorization, modifying their use, or implementing initiatives to improve compliance and behavior. The actual plans are confidential but summaries are publicly available (Toolbox Emissiebeperking 2023). The impact of the agreed ERP is evaluated by identifying temporal and regional trends in the concentration of the concerned substances. The evaluation results are also not published. Imidacloprid, for example, substantially and frequently exceeded threshold values in 2015 (115 of 404 reported concentrations exceeded the RAC (28.5%) with 34 exceedances > 5 × RAC (8%); 173 were above the chronic EQS (JG-MKN) (43%), 63 of which were > 5 × EQS (16%)). The trend observed until 2021 indicates that apart from a few sites, the agreed ERP helped reduce the immission of imidacloprid into surface waters. In 2021, 50 of 498 reported concentrations exceeded the RAC (10%) with 13 exceedances > 5 × RAC (3%), and 84 exceeded the chronic EQS (17%), 18 of which were > 5 × EQS (4%) (values based on information available in the online *Bestrijdingsmiddelenatlas* (Vijver et al. 2008)).

### Use of chemical monitoring for the authorization of plant protection products

As stipulated by Directive 1107/2009/EC, the approval of an a.s. may be subject to the need for monitoring after use (Article 6 (i)) and may be reviewed by the EC at any time, among others when chemical monitoring data indicate that the achievement of the objectives referred to in Article 4(1)(a)(iv) and (b)(i) and Article 7(2) and (3) of Directive 2000/60/EC (WFD) is not secured. Member States may also request a review on these grounds pursuant to Article 44(1) of Directive 2000/60/EC (WFD) and review a national authorization at any time. However, as opposed to the Swiss Plant Protection Product Ordinance, criteria for an obligatory review of an approval or authorization based on chemical monitoring data are not set. As specified in the regulations EU 283/2013 and EU 284/2013 (data requirements for a.s. and PPP, respectively), post-approval monitoring data might be considered for all areas of risk assessment. Again, thresholds or actual procedures are not defined. While EFSA has published several guidance documents on environmental risk assessment, a guidance document on chemical monitoring of surface waters for this purpose is still not available. The issue has been discussed, e.g., in the EFSA guidance on risk assessment of aquatic edge-of-field organisms (EFSA 2013). As a consequence, surface water chemical monitoring data is hardly used in risk assessment at present. None of the member states represented here has appealed to the EC to review the approval of an a.s. due to surface water concentrations.

With regard to national processes, in DK, only national groundwater monitoring data on pesticides has resulted in the withdrawal or restrictions of authorizations of PPP. This has happened several times (Gimsing et al. 2019). Results from surface water monitoring of pesticides for streams from scientific studies have only been used as a qualifier for further surface water screening/monitoring in a few cases. If data are available, they have to be considered in the assessment; however, neither a guidance document nor a defined trigger is available as opposed to groundwater. Within the last reporting period (2004–2012), RAC values were not exceeded in surface water by the substances measured in the national monitoring program in DK (Aarhus Universtet 2015). In NL, chemical monitoring data reported in the Pesticide Atlas is used to evaluate potential exceedances of the RAC and EQS (Vijver et al. 2008). An assessment for the RAC is done for each monitoring location based on the temporal 90-percentile of the measurements in that particular year (see Section 2.3.4.2 in Ctgb (2023)). When the RAC is exceeded, and a causal relation with the proposed use is plausible (i.e. when a statistically significant correlation between threshold exceedance and land use is found based on the correlation analysis as presented in the online Pesticide Atlas (Vijver et al. 2008)), the applicant is requested to submit a further adequate risk assessment to elucidate the probable cause of the exceedance. This might eventually lead to an amendment of the label, restriction, and/or withdrawal/nonauthorization of one or more uses. Exceedances of RAC are also checked upon the application for authorization or extension of the authorization of a PPP. Further details are presented in the case study on emission reduction plans (4.2.1). In CH, authorizations of PPP have already been reviewed based on national monitoring data or new scientific data on adverse effects on non-target organisms. The process was initiated by the risk managers (authorization office) with costs having been covered internally. Recently, the Swiss Water Protection Act and Water Protection Ordinance have been updated with a direct link between chemical monitoring data and PPP and biocides authorization: If chemical monitoring data of surface waters indicate that substances “widely and frequently” exceed the numerical requirements based on ecotoxicological data (acute and chronic aquatic quality standards), the PPP containing that substance will be re-evaluated (Water Protection Act Art. 9 para. 3–6, Water Protection Ordinance Art. 48a). Further application requirements may be imposed on the basis of the review (Water Protection Act Art. 9 para. 4 and 5). Surface water monitoring data are only a trigger to start the re-evaluation process but are not used in the risk assessment. A guidance document is not yet available. In SE, there is a vague link between the authorization process and the national water quality standard regulation according to WFD. So far, only one PPP compound, diflufenican, has been addressed due to exceedances of the threshold value. Although frequently detected and with an arithmetic mean exceeding the EQS (0.01 µg/L; Havs-och vattenmyndigheten 2019) in some waters, there has been only a few samples with concentrations surpassing the RAC used in the authorization process (0.073 µg/L). The risk mitigation activities have been focused on a multi-year information campaign, with the aim to promote sustainable and reduced use of diflufenican in the southern parts of Sweden. In the monitoring period of 2018–2020, during the information campaign, there was no statistically significant decline in diflufenican concentrations as compared to 2015–2017 (Boström and Gönczi 2018). However, measurements in 2021–2022 indicate a decline (personal communication B. Lindstrom, report not yet published), and authorities have decided to continue the campaign. IT has not yet made use of chemical monitoring data for national risk assessment and/or authorization, a trigger for action is not defined.

In FR, the water concentration of a.s. or their degradation products can result in modifications of authorizations. A recent example relates to the assessment of the risk of transfer to groundwater of S-metolachlor metabolites, which has shown unacceptable concentrations of metolachlor-ESA, metolachlor-OXA, and metolachlor-NOA due to exceedances of the quality standard set at 0.1 µg/L. To preserve the quality of water resources, ANSES is initiating a procedure to withdraw authorization for the main uses of PPP containing S-metolachlor. However, the criteria mainly relate to impacts on drinking water, and not to surface water or to aquatic biodiversity protection.

In DE, national groundwater monitoring data on pesticides has resulted in national restrictions of product authorizations and adjustments in risk mitigation measures in a few cases. However, results from surface water monitoring of pesticides for streams from scientific studies and the German monitoring of small streams (KgM, see 4.3.1) have only been used as a qualifier for further surface water monitoring and could, up to now, not be used directly in changing authorizations or product-specific management measures.

In order to use concentrations of a.s. and metabolites in surface waters for the risk assessment of a.s. and PPP, causal links with the use of PPP need to be established. As described in "Information on the origin of pesticides", the EU regulation on pesticide statistics (EC 1185/2009) resulted in rather heterogeneous information with only SE disposing of pesticide usage data on the catchment scale (for four monitoring catchments). The recently adopted regulation (EU) 2023/564 specifying which PPP usage data need to be recorded, and requiring records in electronic format could constitute the basis for EU-wide consistent georeferenced usage data and thus provide well-established links between usage and exposure of surface waters.

#### Case study: small stream monitoring (KgM) in Germany

The “Kleingewässermonitoring (KgM)” was specifically designed to overcome the limitations of the present monitoring with respect to identifying the risks of agricultural pesticides in surface waters. This monitoring of small streams was carried out in Germany in 2018 and 2019 as an activity within the German National Action Plan on the sustainable use of PPP. More than 100 small stream sections were investigated, featuring a full gradient of agricultural land use in the catchment and as few other sources of pesticides as possible focusing on diffuse inputs from agriculture (avoiding wastewater treatment plants if possible, urban areas < 5% (Liess et al. 2022, Liess et al. 2021). In addition to regular grab sampling, rain event-driven sampling was performed to capture transient pesticide peak concentrations entering streams primarily via runoff. Data on PPP usage in the upstream catchment area of monitoring sites were made available after several court decisions (see 4.3) and were compared to the measured pesticide concentrations in the streams. Based on this use data, the occurrence of single pesticides could be exemplarily linked to their agricultural use and even partially to individual PPP applications (Liess et al. 2023).

The monitoring revealed exceedances of RACs in 80% of monitoring sites and 60% of event-driven samples (Liess et al. 2021). Primary reasons for this non-compliance with regulatory goals related to PPP authorization (concentrations in the field below RAC) were identified to lie within (i) the inertia of the risk assessment where re-evaluation of a.s. and subsequently PPP is generally intended only every 10–15 years, but PPP may remain authorized for years even if the current state of knowledge suggests unacceptable environmental risks for the authorized use; (ii) an overestimated efficacy of risk management measures (Klein et al. 2023; Vormeier et al. 2023); (iii) the limitation of the environmental risk assessment to review the use of a single PPP during authorization and thereby neglecting other uses of the same PPP in the same catchment and pesticides mixtures typically occurring in reality.

For surface water authorities, the KgM monitoring data did serve as a qualifier for further monitoring activities: Some of the pesticides identified as risk drivers are to be included in the national list of RBSP and hereby become part of routine monitoring in the future.

Attempts to feed monitoring findings back into the authorization of PPP on a national level concentrated on the re-evaluation of authorizations older than 10 years or imposing stricter risk mitigation for specific PPPs. These approaches would reflect amendments to existing authorizations and are legally covered by Art. 44 EC 1107/2009 but have not yet been taken up by the competent authority for authorizing PPPs in Germany (Federal Office of Consumer Protection and Food Safety).

Even if the monitoring results were plausible (similar pollution levels observed in CH and NL), and the source of pollution was attributed to agricultural pesticide use, some stakeholders requested that the monitoring data should not be used for regulating PPP if contributions of point source pollution, misuse or ignorance of good agricultural practice cannot be fully ruled out. This raises the need to decide whether wide and frequent threshold exceedances alone should trigger a re-evaluation of respective PPP authorizations and which degree of certainty for the causal relation between pesticide threshold exceedances and its use as PPP is needed to withdraw or amend PPP authorizations according to EU legislation.

## Discussion—comparison of chemical monitoring programs

The key indicators characterizing the monitoring programs presented in the previous sections are contextualized regarding their suitability for different purposes in this section. When it comes to the analytical capacities to measure pesticides in surface water, these have substantially increased over the last decade. However, for routine monitoring, there still are gaps to be closed in most countries, especially for the group of pyrethroid insecticides. On the temporal and spatial scale, the monitoring concepts are very heterogeneous as is the usage of the monitoring data in the different countries.

This heterogeneity originates partially from differing goals of the monitoring programs, partially from different hydrology determining the timing and duration of peak concentrations (fast-flowing streams versus slowly streaming canals/ditches), and to some degree also from historically established traditions. Depending on the goals a monitoring strategy is more or less suited to answer the respective questions (Table 4). For detecting whether surface water is polluted from a continuous or diffuse source, taking grab samples at fixed intervals is a suitable and more cost-effective strategy than the other four strategies listed in Table 4. However, for non-continuous sources, other strategies are clearly better suited. Their suitability depends on the goal of the campaign and scientific or regulatory focus. Event-driven samples were chosen in the German “Kleingewässermonitoring” in order to detect peak concentrations which can be compared to RACs (Liess et al. 2021). Time-proportional samples averaging 2 weeks and 3.5 days have, for example, been chosen in CH as they are most suited for comparisons with chronic and acute quality criteria, respectively (Ashauer et al. 2020). Flow-proportional samples allow for determining changes in loads and, if usage data are available, calculating loss rates which can be compared to known loss rates from controlled experiments (e.g., Burgoa and Wauchope (1995)). Finally, high-resolution sampling is useful to gain detailed information on the actually occurring peak concentrations and the underestimation of other monitoring strategies (la Cecilia et al. 2021; Lefrancq et al. 2017; Leu et al. 2004)). Furthermore, high-resolution sampling allows identifying by which pathway the a.s. reached surface water, e.g., by rain-driven run-off or by a point-source of a farm-yard. An important factor to consider for routine monitoring is the costs (first line of Table 4). If a maximal spatial coverage is pursued, then the lower costs of grab samples can be a plus. For the same reasons, passive sampling methods were evaluated and are now officially accepted in FR (Mathon et al. 2022). Another time-integrating sampler like the recently developed TIMFIE (Jonsson et al. 2019) might be a further option to lower costs. This is an active, low-cost sampling device for the quantitative determination of organic micropollutants in whole water.

**Table 4:**
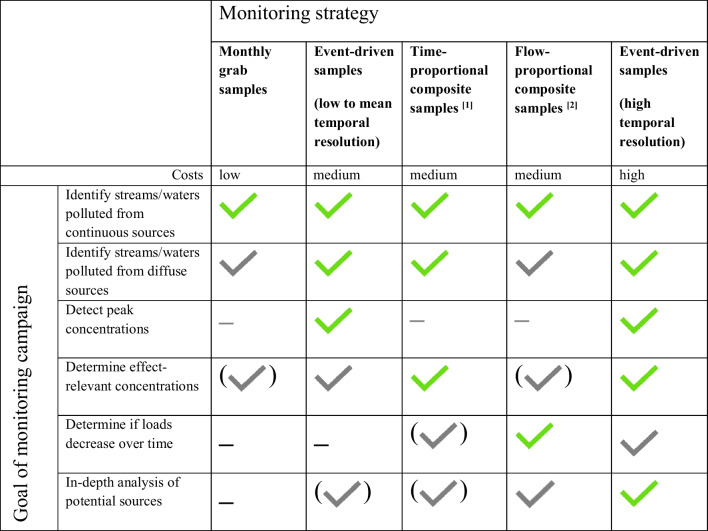
Different goals of monitoring and suitable monitoring strategies. Green checkmarks: ideally suited; grey checkmarks: suited; grey in brackets: limited suitability; dash: not suited

^[1]^1–2 weeks in SE, 2 weeks for chronic quality criteria, and 3.5 days for acute quality criteria in CH

^[2]^In SE, 3 subsamples, but where time from the first to the third sample ranges from 15 min to 24 h depending on changes in flow compared to baseflow criteria)

Due to the high interannual variation, trend detection remains a particular challenge and in many cases is based on the discontinued use of a.s. which are not approved anymore (Chow et al. 2020, 2023). It is therefore understandable that in the context of the sustainable use directive (SUD), apart from NL, SE, and CH, progress is mainly determined with calculated indicators. It should be the aspiration of monitoring programs to complement calculated indicators with real monitoring data to evaluate the success of water protection and agricultural policies. In SE, a toxicity index is calculated, based on monitoring data and national WQOs, to evaluate the progress of the national action plan for SUD (Jordbruksverket 2022). As to the authorization of PPP, comparing monitoring data from countries with similar geographic and climatic conditions can be meaningful when the use of the PPP containing the specific a.s. is similar and if the monitoring programs are designed to facilitate linking measured environmental concentrations to the specific uses causing the exceedance. Analogous to the mutual recognition of PPP authorizations among EU member states of the same zone according to Art. 44 1107/2009/EC, mutual transferability of monitoring data may be justified. Notably, the EU Working Group GWN for Regulators is working to identify vulnerable soils for the monitoring of pesticides. This is aimed to increase harmonization in monitoring data between countries. Similar efforts are made in the EU Working Group Chemicals for the chemical monitoring of surface waters under the WFD.

## Conclusions

The expansion of the possibilities of chemical analytics makes it possible today to cover almost all relevant pesticides in surface waters although there still are substantial gaps in routine monitoring due to practical constraints. The present study shows that while the monitoring programs in different European countries are currently converging in terms of chemical coverage, there are different strategies in terms of temporal and spatial coverage. Some countries put the emphasis on a high spatial coverage in order to represent as many geographical sites and crop areas as possible while other countries maximize the temporal coverage with the goal to cope with the highly dynamic nature of pesticide concentrations and to be in a better position to detect trends.

The experiences in using monitoring data for pesticide regulation from the surveyed countries underline the need for (i) agreed criteria for requirements and quality assessment of monitoring data, (ii) guidance on using monitoring data for regulatory analyses, and (iii) a commitment of member states to withdraw or amend PPP authorizations in the light of monitoring data indicating that regulatory goals for a PPP active ingredient are not met. The long-term goal could be to establish comparable data across Europe to assess the success of the Green Deal. As the national monitoring programs have historically grown, this is a major task. However, it would allow to more efficiently couple the environmental monitoring programs with the regulation and management of chemicals at the European level, thereby allowing to take measures where they are most needed. The success of policies to reduce pesticide pressures on surface waters would further benefit from the usage and coupling of measured and calculated indicators required to effectively detect trends and from the availability of data on pesticide usage at the catchment level. Currently, the Working Group Chemicals of the European Commission is developing guidance for monitoring under the WFD with the aim to describe various good practices for monitoring substances with variable exposure patterns like PPP in accordance with the WFD requirements. Ideally, these good practices would also take into consideration the potential use of chemical monitoring data for risk assessment of PPP.

### Supplementary information

Below is the link to the electronic supplementary material.

Supplementary file1 (DOCX 72 KB)

## Data Availability

All data collected for the present study are included in the supporting information. No further datasets or other materials are necessary.
