# Supplementary material for: Linking chemical surface water monitoring and pesticide regulation in selected European countries
Source: Environ Sci Pollut Res Int. 2024 Jun 12;31(30):43432–50. doi: 10.1007/s11356-024-33865-y (PMC11222191; doi:10.1007/s11356-024-33865-y)
Supplement: Supplementary file 1 — Supplementary file1 (DOCX 72 KB) [file 11356_2024_33865_MOESM1_ESM.docx]

**Linking chemical surface water monitoring and pesticide regulation in selected European countries**

Names of the Authors:

Simon Spycher^1^, Dennis Kalf^2^, Joost Lahr^3^, Mikaela Gönczi^4^, Bodil Lindström^4^, Emanuela Pace^5^, Fabrizio Botta^6^, Nolwenn Bougon^7^, Pierre-François Staub^7^, Kristina Lotte Hitzfeld^8^, Oliver Weisner^8^, Marion Junghans^9^, and Alexandra Kroll^9*^

^1^Daten Spycher GmbH, 8057 Zürich, Switzerland.

^2^Rijkswaterstaat, Ministry of Infrastructure and Water Management, PO Box 2232,

3500 GE Lelystad, the Netherlands

^3^National Institute of Public Health and the Environment, PO Box 1, 3720 BA Bilthoven, the Netherlands

^4^Department of aquatic sciences and assessment, SLU Centre for pesticides in the environment, Swedish University of Agricultural Sciences, P.O. Box 7050, SE-75007 Uppsala, Sweden

^5^Italian Institute for Environmental Protection and Research (ISPRA), 00144 Rome, Italy

^6^Unit of pesticidovigilance, ANSES, Maisons-Alfort, France

^7^French Biodiversity Agency – OFB, 94300 Vincennes, France.

^8^German Environment Agency (UBA), 06844 Dessau-Roßlau, Germany.

^9^Swiss Centre for Applied Ecotoxicology, 8600 Dübendorf, Switzerland.

^*^Corresponding author: [alexandra.kroll@oekotoxzentrum.ch](mailto:alexandra.kroll@oekotoxzentrum.ch)

- 1. Questionnaire

**Topic 1: National Monitoring of Pesticides in Small Water Bodies**

**1. Characteristics of Pesticide Monitoring Programs**

1.1 Is the legislation and/or guidance on pesticide monitoring implemented on the national level or on the regional level (e.g. “Bundesland” or “Kanton”)?

1.2 Are small water bodies included?

1.3 If feasible and applicable, please indicate for the national monitoring:

1. Type of samples: In case grab samples are the main type, are they triggered by events or by time interval? In case composite samples are the main type, are they time-proportional or flow proportional?
2. Sampling intervals (i.e., how many grab samples a month), temporal resolution (i.e., time period of composite samples).
3. Number of measured active substances, number of measured metabolites
4. Criteria for including compounds in the list of measured substances. If available please upload a list of the substances measured including a suitable ID like CAS-or CIPAC-Number (Collaborative International Pesticides Analytical Council)
5. Number of sampling sites (fixed or variable locations)
6. Surface of arable land. Surface of other crops with relevant pesticide usage (i.e. orchards, vineyards, berries, other) in ha
7. Size/size class of catchments (e.g., share of catchments < 10 km^2^, 10-100 km^2^ and > 100 km^2^ or stream order or according to mean discharge)
8. **Administration of Monitoring Programs**
   1. How are the costs for the monitoring campaigns covered? Is there a cost allocation to plant protection product sales based on substance properties or unit dose?
   2. Is surface water monitoring data generally collected in a national database?
   3. If yes, is the database publically available or is access restricted?
9. **Additional Information Improving Interpretability**
   1. Are WWTP markers measured and if yes, which ones (e.g. caffeine, sucralose or other)?
   2. Are pesticide monitoring data compared with plant protection usage data and if yes, for which goals (e.g., calculation of loss rates)?
   3. Are there approaches to distinguish the usage for plant protection from the usage as biocide and veterinary medicines (e.g., quantitative data on urban use of biocides)?
10. **Goals and Philosophy of Monitoring Strategies**
    1. Are pesticide monitoring data mainly used to identify peak concentrations (and hence exceedances of RAC) or rather to quantify in the progress achieved by implementing risk reduction measures?
    2. Do you agree that the first goal, i.e., identifying peak concentrations, is rather achieved by event based sampling and the latter, i.e., the quantification of progress, rather by composite samples?
    3. Which sampling strategy is used in your country and for which reasons?
    4. Which threshold value(s) are monitoring data compared to (e.g. EQS, RAC,…)? Are the same threshold values used for all type of water bodies (i.e., small streams and rivers or lakes)?
11. **Possible Benefits of Harmonization**
    1. Do you think that the currently available surface water monitoring data allow meaningful comparisons between European countries concerning the quality of surface waters?
    2. Do you think that the use of monitoring data from countries with similar geographic and climatic conditions would be meaningful for the authorisation of plant protection products?
    3. What is your opinion on harmonizing sampling strategies for monitoring of small water courses across Europe? Would that allow for Europe-wide comparison of chemical water quality?

**Topic 2: Authorisation of Plant Protection Products**

1. **Adaptations on Member State Level**
   1. Did your country specify national procedures for environmental risk assessment that differ from the EFSA guidance?
   2. If yes, are these considered legitimate or have they been challenged?
   3. Does your country publish a list of national RACs? If yes, would you be able to share these (possibly together with the origin, i.e. species/endpoint and assessment factor)?
   4. Has your country ever filed a request to the EC to review an approval based on Art. 21/Art. 44 (EC 1107/2009)? If yes, who is responsible for this process and who covers the costs?
2. **Risk Mitigation Measures (RMM)**
   1. Which RMM for surface waters from the table below are applied by the farmers according to your knowledge?
   2. Are there national programs defining goals for the total length or percentage of watercourses with riparian buffer strips (e.g., from remote sensing data) and the type (continuous/discontinuous, vegetated)?
   3. If conservation tillage is an RMM in your country, are there data on the fraction of arable land with conservation tillage? Is there a national and binding definition of conservation tillage, e.g. specifying the percentage of crop residues to be left on the field or the type of machinery that can be used?
   4. Are there national rules on the dimensions of and obligations for riparian buffer strips and if so, can you briefly describe how they are formulated?
   5. Is the implementation of risk management measures verified?
3. **Use of Monitoring for the Authorization of Plant Protection Products**
   1. Do you collect plant protection product usage data, e.g., for comparison with surface monitoring data?
   2. Are national surface water monitoring data used for the authorisation of plant protection products?
   3. Question specific to DK: How are the monitoring data on surface water from the NOVANA program for the risk assessment of aquatic organisms?
   4. In case surface water monitoring data is used for authorisation, is there national guidance on how monitoring data are to be used?
   5. Is there a trigger for when an authorisation needs to be adapted based on exceedances?
   6. Do you think that monitoring data can be used to restrict PPP uses under the SUD (Sustainable Use Directive (2009/128/EC)?
   7. Data
      1. Data on Land use
         1. FAO-Data on agricultural land use

Table A1: Land use in the year 2020 data retrieved from FAOSTAT ^[1]^. Cropland is the sum of arable land and land under permanent crops.

| **Country** | **Agricultural land**  **[ha]** | **Arable land**  **[ha]** | **Land under permanent crops**  **[ha]** | **Cropland**  **[ha]** |
| --- | --- | --- | --- | --- |
| Denmark | 2’619’987 | 2’370’930 | 26’652 | 2’397’582 |
| France | 28’553’754 | 17’956’560 | 1’013’980 | 18’970’540 |
| Germany | 16’595’000 | 11’664’000 | 198’000 | 11’862’000 |
| Italy | 12’999’000 | 6’831’000 | 2’427’000 | 9’258’000 |
| Netherlands | 1’814’450 | 1’004’830 | 37’210 | 1’042’040 |
| Sweden | 3’005’540 | 2’538’550 | 3’480 | 2’542’030 |
| Switzerland | 1’504’214 | 399’842 | 25’196 | 425’037 |

^[1]^ <https://www.fao.org/faostat/en/#data/RL> Data > Land, Inputs and Sustainability > Land > Land use

- - - 1. Country specific data

Table A2: Land use in the year 2020 data retrieved from FAOSTAT. Cropland is the sum of arable land and land under permanent crops.

| **Parameter** | **Agricultural land**  **[ha]** | **Arable land**  **[ha]** | **Land under permanent crops**  **[ha]** | **Cropland**  **[ha]** |
| --- | --- | --- | --- | --- |
| France ^[1]^ | 28’449’413 | 14’351’615 | 1’275’160 | 15’626’775 |
| Switzerland ^[2]^ | 1’504'214 ^[3]^ | 274'449 ^[4]^  401'872 ^[5]^ | 20’833 | 295’282 ^[4]^  422'705 ^[5]^ |

^[1]^ <https://agreste.agriculture.gouv.fr/agreste-saiku/?plugin=true&query=query/open/W0020#query/open/W0020> Limited to France métropolitaine

^[2]^ Federal Office of Agriculture (FOAG) 2022: Agrarbericht, [www.agrarbericht.ch](http://www.agrarbericht.ch)

^[3]^ Agricultural land including alpine farming

^[4]^ Arable land without ley (meadow on arable land) and ^[5]^ Arable land including ley

Note: Data show some deviations from FAO-stat, but confirm that value for cropland is better reference for determining spatial coverage.

- - 1. Data on Risk Mitigation Measures
       1. Denmark

| Type of RMM | **RMM imposed for pesticide product authorization*** (Yes/No) For which type of entry path  (Drift: Dr, Runoff: Ro, Drainage: Dn) | **Selectable RMM#** (e.g. as part of a point system) (Yes/No) | **Method of control** (e.g., Remote Sensing, On-site control, etc.) | **Quantitative data on frequency of use available** (e.g. fraction of land use with conservation tillage) |
| --- | --- | --- | --- | --- |
| a. Riparian buffer strips | Dr |  |  |  |
| b. Conservation tillage |  |  |  |  |
| c. Drift reduction (e.g. drift reducing nozzles or other measures) | Dr |  |  |  |
| d. Contour cropping |  |  |  |  |
| e. Microdams |  |  |  |  |
| f. Cover plants ("Untersaat") |  |  |  |  |
| g. Groundwater protection measures with beneficial effects for surface waters | Dn |  |  |  |
| h. In-field buffers | Dr |  |  |  |
| i. [Others] |  |  |  |  |

* This refers to RMM which need to be applied under all circumstances.

# This refers to compulsory RMM selectable from a list of options.

- - - 1. France

| Type of RMM | **RMM imposed for pesticide product authorization*** (Yes/No) For which type of entry path  (Drift: Dr, Runoff: Ro, Drainage: Dn) | **Selectable RMM#** (e.g. as part of a point system) (Yes/No) | **Method of control** (e.g., Remote Sensing, On-site control, etc.) | **Quantitative data on frequency of use available** (e.g. fraction of land use with conservation tillage) |
| --- | --- | --- | --- | --- |
| a. Riparian buffer strips | Yes, the Dr for all pesticides and for some the Dr and /or the Dn |  |  |  |
| b. Conservation tillage | Only planned for nitrate vulnerable areas |  |  |  |
| c. Drift reduction (e.g. drift reducing nozzles or other measures) | "Products can only be used when the wind is less than or equal to 3 on the Beaufort scale. They cannot be used when the intensity of rainfall is greater than 8 mm per hour, at the time of treatment. |  |  |  |
| d. Contour cropping | (article 2 of the order of 4/05/2017) |  |  |  |
| e. Microdams | Materials limiting drift are mandatory for some pesticides. They are also mandatory associated with permanent vegetation devices to reduce the no-treatment zones (for example from 20 meters to 5 meters) |  |  |  |
| f. Cover plants ("Untersaat") | " |  |  |  |
| g. Groundwater protection measures with beneficial effects for surface waters |  |  |  |  |
| h. In-field buffers |  |  |  |  |
| i. [Others] | Only planned for nitrate vulnerable areas |  |  |  |

* This refers to RMM which need to be applied under all circumstances.

# This refers to compulsory RMM selectable from a list of options.

- - - 1. Germany

| Type of RMM | **RMM imposed for pesticide product authorization*** (Yes/No) For which type of entry path  (Drift: Dr, Runoff: Ro, Drainage: Dn) | **Selectable RMM#** (e.g. as part of a point system) (Yes/No) | **Method of control** (e.g., Remote Sensing, On-site control, etc.) | **Quantitative data on frequency of use available** (e.g. fraction of land use with conservation tillage) |
| --- | --- | --- | --- | --- |
| a. Riparian buffer strips | Yes / Ro ^1^ |  | On site | Yes/No ^3^ |
| b. Conservation tillage | Yes / Ro ^1^ |  | On site but no controllable coverage level established | Yes ^4^ |
| c. Drift reduction (e.g. drift reducing nozzles or other measures) | Yes / Dr |  | On site | No |
| d. Contour cropping | No |  |  | No |
| e. Microdams | No (under discussion) |  |  | No |
| f. Cover plants ("Untersaat") | No |  |  | No |
| g. Groundwater protection measures with beneficial effects for surface waters | Yes |  | On site | No |
| h. In-field buffers | buffer strips ^2^, limitation of application rates |  |  | No |
| i. [Others] | No (under discussion) |  |  | No |

* This refers to RMM which need to be applied under all circumstances.

# This refers to compulsory RMM selectable from a list of options.

^1^ One of these options is mandatory if the risk assessment indicates the need to reduce runoff

^2^ to reduce Ro into surface water and subsequent infiltration into groundwater

^3^ There used to be an indicator for the national action plan (buffer stripes along vulnerable surface waters), not monitored after 2016

^4^ Statistisches Bundesamt publishes fraction of arable land with conservation tillage

- - - 1. Italy

| Type of RMM | **RMM imposed for pesticide product authorization*** (Yes/No) For which type of entry path  (Drift: Dr, Runoff: Ro, Drainage: Dn) | **Selectable RMM#** (e.g. as part of a point system) (Yes/No) | **Method of control** (e.g., Remote Sensing, On-site control, etc.) | **Quantitative data on frequency of use available** (e.g. fraction of land use with conservation tillage) |
| --- | --- | --- | --- | --- |
| a. Riparian buffer strips | Dr, Ro |  |  |  |
| b. Conservation tillage | Ro |  |  |  |
| c. Drift reduction (e.g. drift reducing nozzles or other measures) | Dr |  |  |  |
| d. Contour cropping | Ro |  |  |  |
| e. Microdams | No |  |  |  |
| f. Cover plants ("Untersaat") | Ro |  |  |  |
| g. Groundwater protection measures with beneficial effects for surface waters | Unclear |  |  |  |
| h. In-field buffers | Ro |  |  |  |
| i. [Others] | Vegetated ditch |  |  |  |

* This refers to RMM which need to be applied under all circumstances.

# This refers to compulsory RMM selectable from a list of options.

- - - 1. The Netherlands

| Type of RMM | **RMM imposed for pesticide product authorization*** (Yes/No) For which type of entry path  (Drift: Dr, Runoff: Ro, Drainage: Dn) | **Selectable RMM#** (e.g. as part of a point system) (Yes/No) | **Method of control** (e.g., Remote Sensing, On-site control, etc.) | **Quantitative data on frequency of use available** (e.g. fraction of land use with conservation tillage) |
| --- | --- | --- | --- | --- |
| a. Riparian buffer strips | No |  |  |  |
| b. Conservation tillage | No |  |  |  |
| c. Drift reduction (e.g. drift reducing nozzles or other measures) | Yes |  |  |  |
| d. Contour cropping | No |  |  |  |
| e. Microdams | No |  |  |  |
| f. Cover plants ("Untersaat") | No |  |  |  |
| g. Groundwater protection measures with beneficial effects for surface waters | Yes |  |  |  |
| h. In-field buffers | Yes |  |  |  |
| i. [Others] | No |  |  |  |

* This refers to RMM which need to be applied under all circumstances.

# This refers to compulsory RMM selectable from a list of options.

- - - 1. Sweden

| Type of RMM | **RMM imposed for pesticide product authorization*** (Yes/No) For which type of entry path  (Drift: Dr, Runoff: Ro, Drainage: Dn) | **Selectable RMM#** (e.g. as part of a point system) (Yes/No) | **Method of control** (e.g., Remote Sensing, On-site control, etc.) | **Quantitative data on frequency of use available** (e.g. fraction of land use with conservation tillage) |
| --- | --- | --- | --- | --- |
| a. Riparian buffer strips | Yes, but only for one product, Runoff. Previously included as an option for risk mitigation in the authorisation process for PPPs. Since 2016 the runoff-scenario has been excluded from the risk assessments and so also this condition of use. | No | No | No |
| b. Conservation tillage | No | No | No | No |
| c. Drift reduction (e.g. drift reducing nozzles or other measures) | Yes, with the Helper ”Mitigating spray drift in Sweden” https://www.sakertvaxtskydd.se/bibliotek/ | No | No | No |
| d. Contour cropping | No | No | No | No |
| e. Microdams | No | No | No | No |
| f. Cover plants ("Untersaat") | No | No | No | No |
| g. Groundwater protection measures with beneficial effects for surface waters | No | No | No | No |
| h. In-field buffers | No | Yes in CAP-system but not specifically for PPPs rather for erosion and nutrients. | No | No |
| i. [Others] | No | No | No | No |

* This refers to RMM which need to be applied under all circumstances.

# This refers to compulsory RMM selectable from a list of options.

- - - 1. Switzerland

| Type of RMM | **RMM imposed for pesticide product authorization*** (Yes/No) For which type of entry path  (Drift: Dr, Runoff: Ro, Drainage: Dn) | **Selectable RMM#** (e.g. as part of a point system) (Yes/No) | **Method of control** (e.g., Remote Sensing, On-site control, etc.) | **Quantitative data on frequency of use available** (e.g. fraction of land use with conservation tillage) |
| --- | --- | --- | --- | --- |
| a. Riparian buffer strips | Y | Y (Dr, Ro) | On-site | Y |
| b. Conservation tillage | N | Y (Ro) | Y | Y |
| c. Drift reduction (e.g. drift reducing nozzles or other measures) | N | Y (Dr) | Y (cf. footnote) | Partially  (cf. footnote) |
| d. Contour cropping | N | N | - | - |
| e. Microdams | N | Y (Ro) | Y | - |
| f. Cover plants ("Untersaat") | N | N (cf. footnote) | Y | Partially  (cf. footnote) |
| g. Groundwater protection measures with beneficial effects for surface waters | Y | N | Y | N |
| h. In-field buffers | N | Y (Ro) | N | N |
| i. [Others] | cf. Footnote |  |  |  |

* This refers to RMM which need to be applied under all circumstances.

# This refers to compulsory RMM selectable from a list of options.

- 1. Evaluations
     1. Specific tables compiling information from the questionnaire complemented with public government reports

Table A3: Key indicators of pesticide monitoring strategies

| **Country** | **Mean Number of a.s. per sample** | **Ref.** | **Average number of samples per year and site** | **Ref.** | **Number of sites per year** | **Ref.** |
| --- | --- | --- | --- | --- | --- | --- |
| Denmark | - | ^[1]^ | 4 | ^[1]^ | 19 | ^[1]^ |
| France | 53 | ^[2]^ | 12 | ^[3]^ | 4000 | ^[3]^ |
| Germany | 80 | ^[4]^ | 4 | ^[5]^ | 1037 | ^[5]^ |
| Germany KGM | 86 | ^[12]^ | 6 | ^[13]^ | 70 | ^[13]^ |
| Italy | 81 | ^[6]^ | 5.16 | ^[6]^ | 1837 | ^[6]^ |
| Netherlands | - | ^[7]^ | 12 | ^[7]^ | 106 | ^[7]^ |
| Sweden | 135 | ^[8]^ | 29 | ^[8]^ | 6 | ^[8]^ |
| Switzerland | 62 | ^[9]^ | 26 | ^[10]^ | 30 | ^[11^ |

^[1]^ Only information on minimum required number of a.s. available. Number of sites per year derived from questionnaire ("95 in a 5 years period") with the assumption that each site is measured every fifth year, i.e., 95/5=19

^[2]^ One metabolite (AMPA) excluded

^[3]^ Answer from questionnaire

^[4]^ Brinke et al. 2017, Section 3.4.3, and Table 4. Value in Table 4 includes a limited number of metabolites. Both values include a high number of compounds that were not authorized any more.

^[5]^ Values taken from Table 2 of of Brinke *et al.* (2017) with number of sites given as total over 11 years. Assumption that on average each site is sampled every third year and thus 3112/3=1037 sites per year resulting in 46295/11 = 4209 samples a year and approximately 4 samples per site and year.

^[6]^ All values from ISPRA (2022) with the average number of a.i. from p. 19 and number of samples and sites from Tab. 4.1 for the year 2020 (9477 samples taken from 1837 sites and thus an average 5.16 samples per site and year)

^[7]^ Maximum number. According to Deltares (2021) Annex H.7 Table B.1 number of compounds determined and number of samples depends on land use with lowest values for grassland and fodder crops and higher values for crops with frequent pesticide use like vegetables, flower bulbs, greenhouses.

^[8]^ Answers from questionnaire: 4 sites with weekly composite samples (100 subsamples a week) from April-October/November, i.e., 116 samples (12’600 subsamples, respectively) of which 2 sites also is sampled with two-weekly composite samples (100 subsamples during two weeks) from December-March, i.e., 2 x 9 samples = 18 samples (2016 subsamples, respectively), and of which 1 site also have event driven sampling with 24 samples per year, 2 sites with 6 grab samples between May-Jul and 4 samples between Aug-Nov, i.e. 20 samples and thus a total of 116+18+24+20 = 178 samples for 6 sites, resulting in an average 29 samples/site.

^[9]^ Answers from questionnaire: Some samples are special cases with Glyphosate measurements only. Exclusion of those samples increases mean number measured PPP to 64.

^[10]^ Answers from questionnaire: Total number of sites is 38, but 8 not used for evaluation of pesticide trends due to major influence of urban sites. Apart from the 38 sites of the national NAWA-network there is a substantial number of additional sites measured by cantons often in a rotation, i.e., not every year.

^[11]^ Answers from questionnaire: Actual number of samples might be higher as there is a substantial number sites taking 3.5d-composite samples from April-July instead of 2w-composite samples. On the other hand there might be some sites not taking samples in the winter months. The number of 26 samples indicates that on average all sites are sampled all year with 2-week composite samples.

^[12]^ Liess et al. (2022) Table 1.

^[13]^ Liess et al. (2021) Total of 840 samples in two years analysed. Number of streams per year is 70 (including some reference streams without agricultural land use).

**References**

Brinke, M.; Bänsch-Baltruschat, B.; Keller, M.; Szöcs, E.; Schäfer, R. B.; Foit, K.; Liess, M. (2017): Umsetzung des Nationalen Aktionsplans zur nachhaltigen Anwendung von Pestiziden. Bestandsaufnahme zur Erhebung von Daten zur Belastung von Kleingewässern der Agrarlandschaft. UBA-Texte 89/2017, Umweltbundesamt. Dessau-Roßlau, 142 p.

Deltares (2020): Landelijk Meetnet Gewasbeschermingsmiddelen Land- en Tuinbouw, Evaluatie resultaten 2020, Buijs, S., van den Meiracker R., Tamis W., van 't Zelfde, 78 p.

ISPRA (2022): Rapporto nazionale pesticidi nelle acque, Dati 2019-2020, Edizione 2022, 98 p.

Liess M et al. (2021): Pesticides are the dominant stressors for vulnerable insects in lowland streams. Water Research 201, 117262.

Liess *et al.* (2022): Umsetzung des Nationalen Aktionsplans zur nachhaltigen Anwendung von Pflanzenschutzmitteln (NAP) – Pilotstudie zur Ermittlung der Belastung von Kleingewässern in der Agrarlandschaft mit Pflanzenschutzmittel-Rückständen, UBA-Texte 07/2022.
